# Supplementary material for: Patched regulates lipid homeostasis by controlling cellular cholesterol levels
Source: Nat Commun. 2021 Aug 12;12:4898. doi: 10.1038/s41467-021-24995-9 (PMC8361143; doi:10.1038/s41467-021-24995-9)
Supplement: Supplementary file 1 — Supplementary Information [file 41467_2021_24995_MOESM1_ESM.pdf]

Figure S1

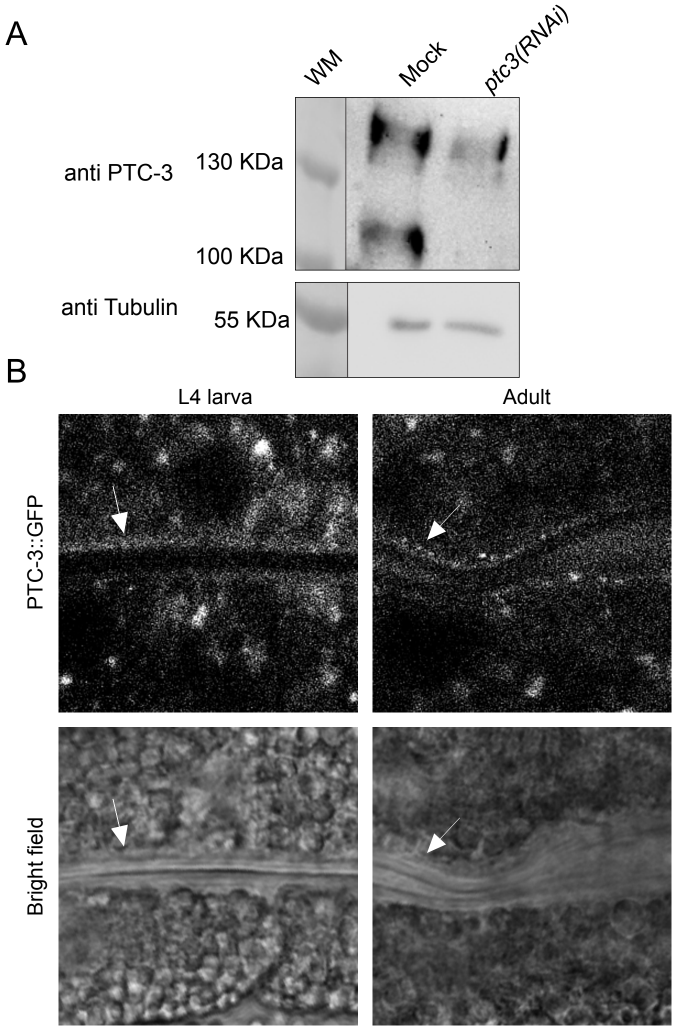

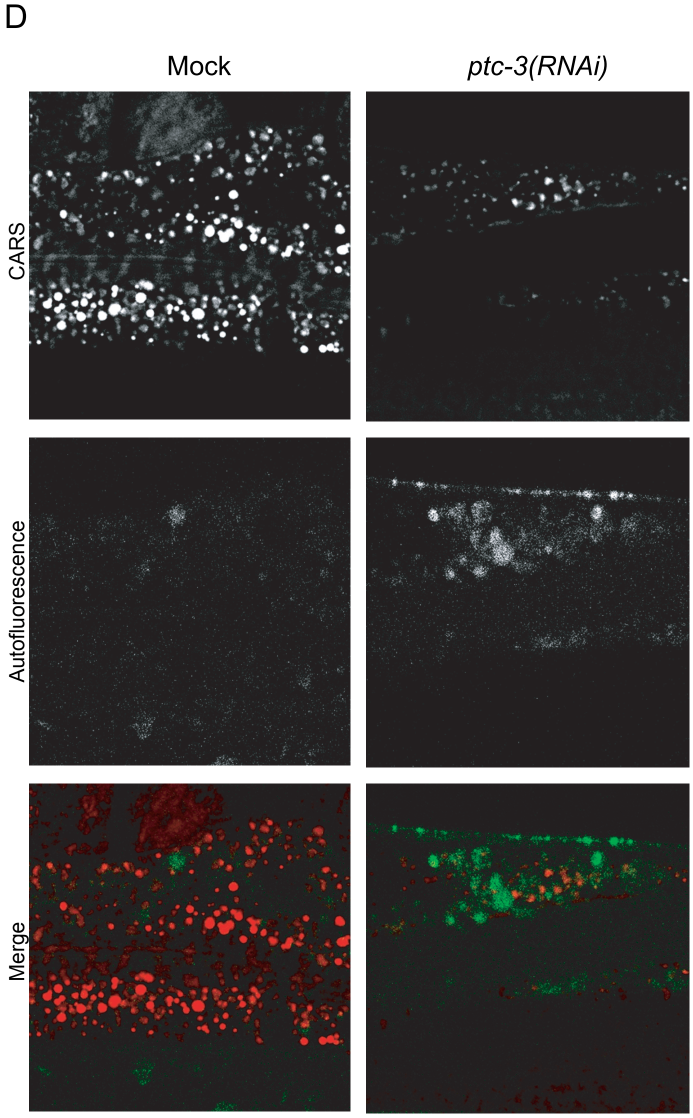

C

Strain \ RNAi	Mock	*pos-1(RNAi)*
N2	55/55	0/28
RNAi$_{gut}$	18/18	28/28

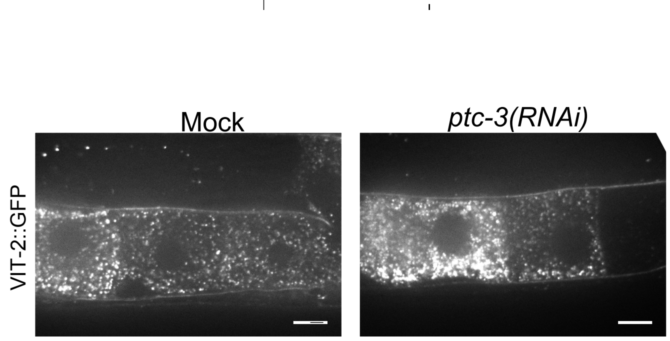

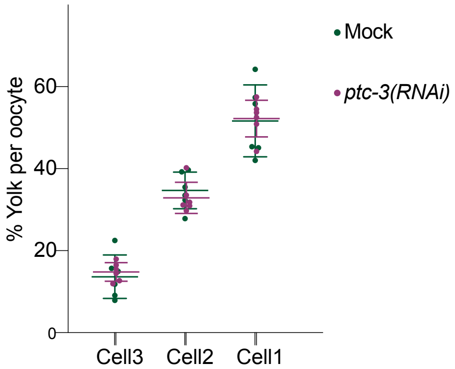

Figure S1. PTC-3 expression pattern. (A) Antibodies against PTC-3 are specific. Immunoblot of lysates of mock or *ptc-3 (RNAi)* treated worms. n=3 (B) PTC-3 gut localization was confirmed with a GFP-tagged PTC-3. Arrows point to the apical intestinal membrane. Scale bars 10 µm n=10 (C) Quantification of the number of N2 or RNAigut animals with living progeny upon mock or *pos-1(RNAi)* treatment. (D) Mock and *ptc-3(RNAi)* treated animals were imaged with CARS microscopy. The signal did not overlap with the autofluorescence of LROs, validating the proper spectral separation of the filters, and that we can detect specifically lipid droplets. Scale bars 10 µm. n=22 (Mock) and 21 *(ptc-3(RNAi))* (E) Yolk content in the oocytes was address by analyzing VIT-2::GFP in the worm oocytes of mock and *ptc-3(RNAi)* after L2 stage treated animals. No difference could be observed between both conditions. (F) Quantification of data shown in (E) Mean and SD error bars n=6. Scale bars; 10 µm. Unprocessed blots and statistical source data are available as source data.

Figure S2

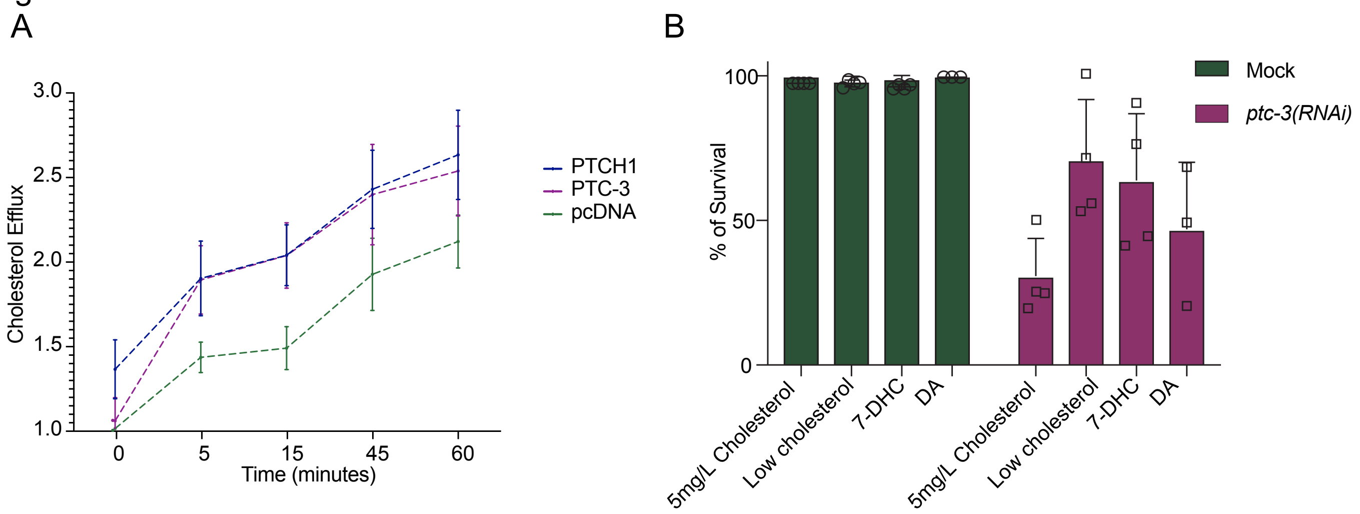

Figure S2. PTCH proteins are cholesterol transporters (A) PTCH1 and PTC-3 expression induced cholesterol efflux from cells. HEK293 expressing PTC-3 or PTCH1 were incubated for 2 hr with 2.5 mM TopFluor® Cholesterol, washed with PBS, and incubated with imaging buffer. After 5, 15, 45, and 60 min, the fluorescence intensity of the media supernatant was measured. The values were normalized against time 0 empty vector. Mean and SEM error bars n=3. (B) Neither 7-DHC nor DA rescued the *ptc-3(RNAi)* phenotype. Quantification of number of *ptc-3(RNAi)* treated worms that reached adulthood when fed from L1 larva under standard cholesterol conditions (5 mg/l), without cholesterol or when cholesterol was replaced with 7-DHC or DA for 3 days. Substitution of 7-DHC or DA did not alleviate the *ptc-3(RNAi)* arrested phenotype over the no cholesterol control. Mean and SEM error bars n=3. Statistical source data are available as source data.

Figure S3

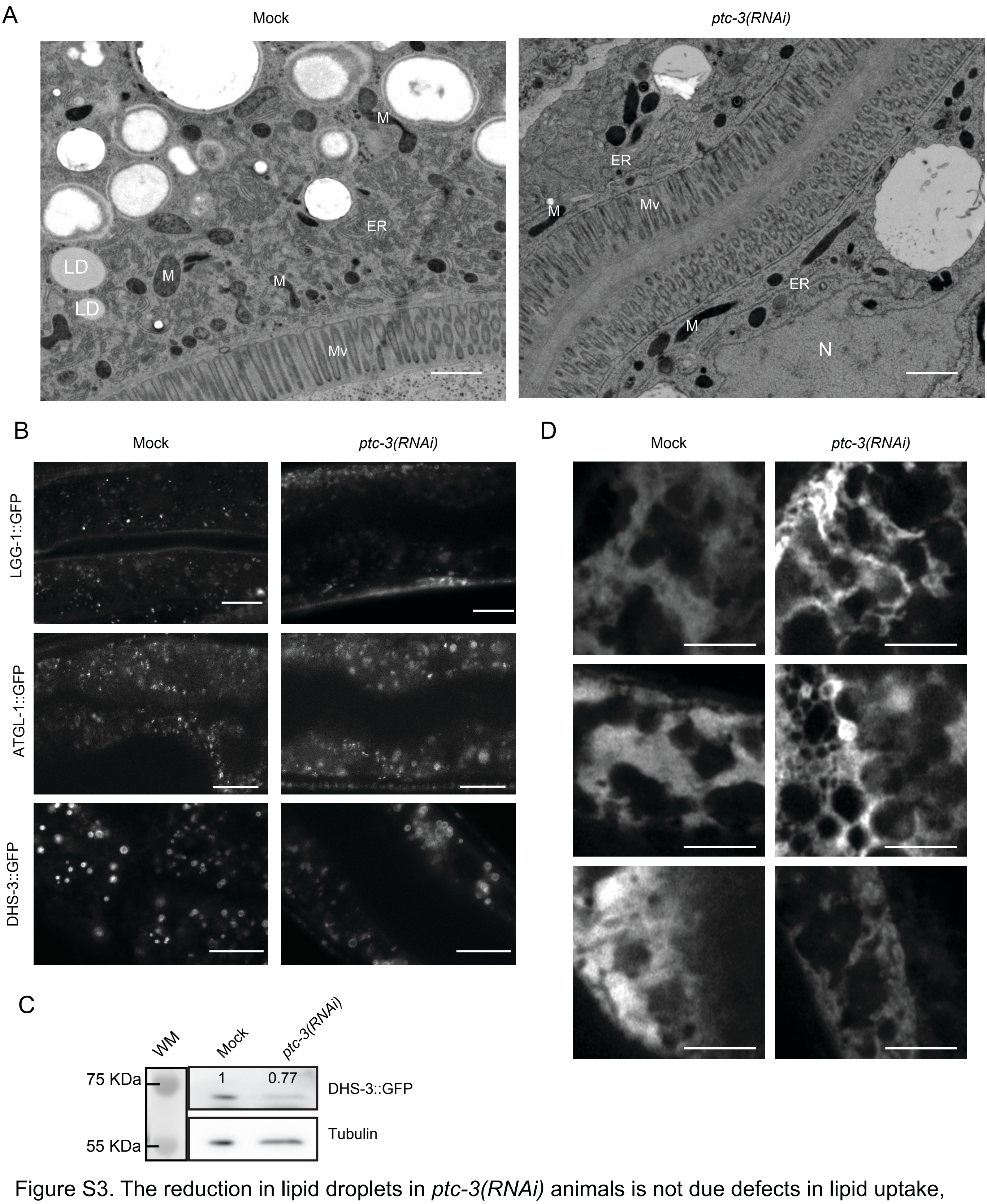

Figure S3. The reduction in lipid droplets in *ptc-3(RNAi)* animals is not due defects in lipid uptake, increased autophagy or lipolysis or reduction of lipid droplet proteins. (A) Electron microscopy images of parts of the intestine of mock and *ptc-3(RNAi)* treated animals. No changes in microvilli or plasma membrane organization were observed. Bars 1 μm n= 4 (mock) and 6 *(ptc-3(RNAi)*) (B) Fluorescence microscopy images of LGG-1::GFP n= 17 (mock) and 9 *(ptc-3(RNAi)*), ATGL-1::GFP n= 22 (mock) and 18 (*ptc-3(RNAi)*) or DHS-3::GFP n= 57 (mock) and 31 (*ptc-3(RNAi)*) in mock or *ptc-3(RNAi)* treated animals showing no differences between both conditions (C) Immunoblot of lysates of mock or *ptc-3 (RNAi)* treated DHS-3::GFP worms showing just a slight reduction of DHS-3 level upon *ptc-3 (RNAi)* treatment n=3. Unprocessed blots are available as source data. (D) ER morphology appears to be altered upon *ptc-3(RNAi)*. Light microscopy images of intestinally expressed TRAM-GFP. Images suggest morphological alterations in the ER in *ptc-3(RNAi)* animals. n= 14 (mock) and 10 *(ptc-3(RNAi)*) from 3 independent experiments. Scale bar 5 μm.

Figure S4

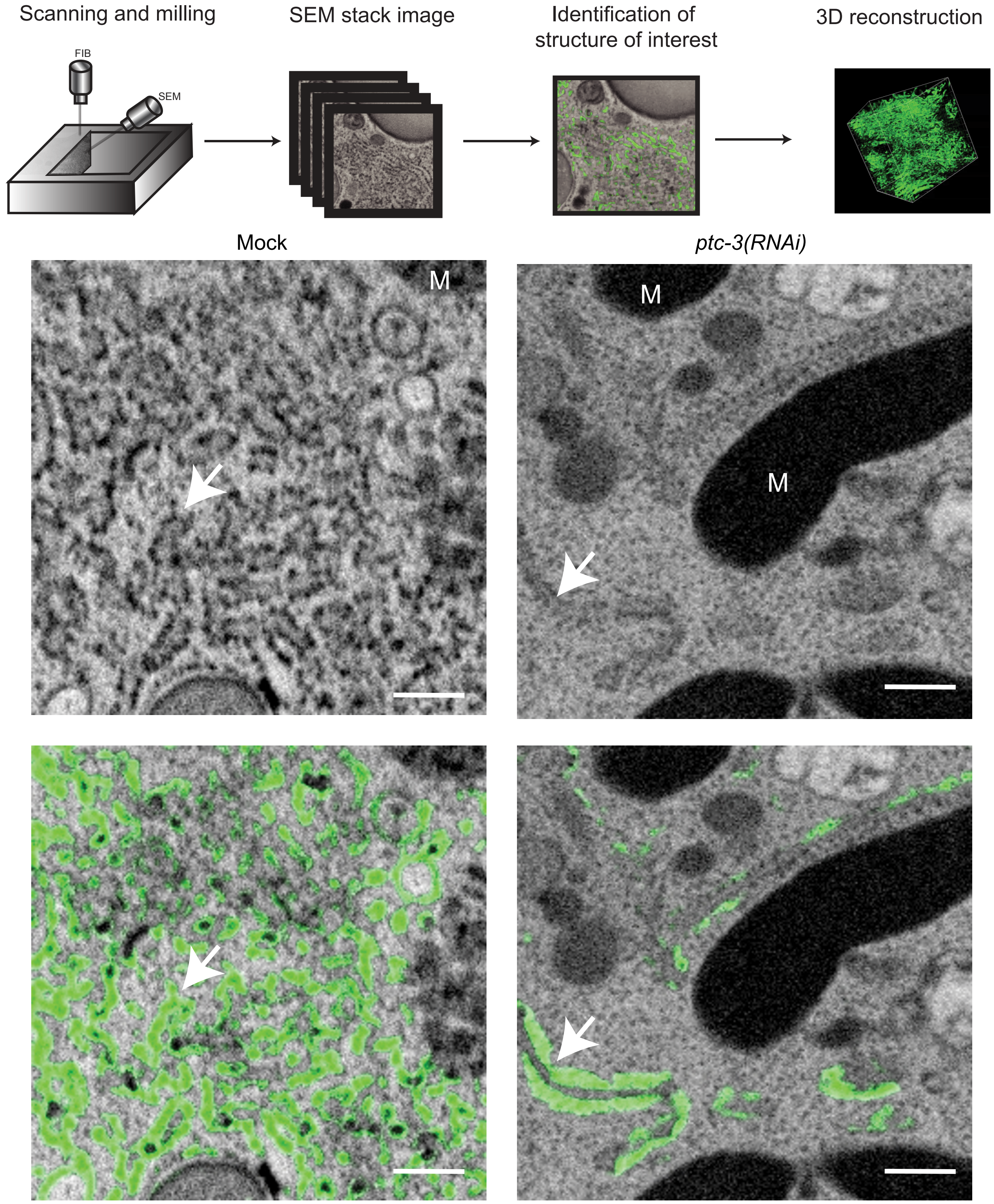

Figure S4. FIB-SEM analysis of intestinal cell reveals sheet-like ER in *ptc-3(RNAi)* animals. (A) Workflow of FIB-SEM analysis. After TEM analysis a region of interest was chosen, SEM images were acquired followed by milling steps. A SEM Z-stack was generated, and identification of the ER was done by iLastik training. (B) Representative images showing automatic ER identification in green by iLastik after machine learning training sessions. n=3 from 1 animal per condition Scale bars 200 nm.

Figure S5

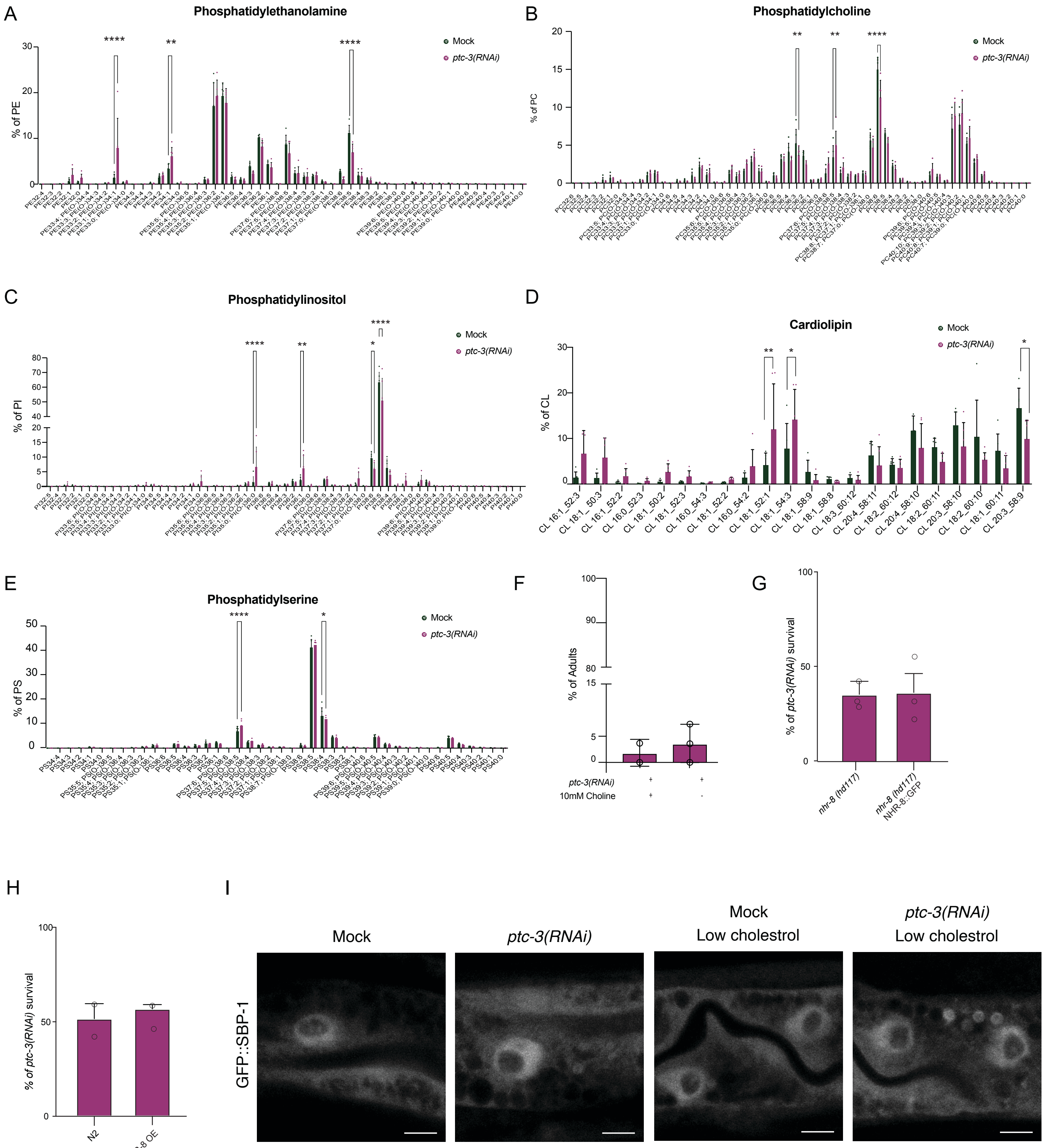

Figure S5. Acyl chain saturation levels but not NHR-8 nor SBP-1 play a role in *ptc-3(RNAi)*. (A-E) Comparison of the distribution of different lipid species upon *ptc-3(RNAi)*. Individual data points, mean values and error bars are represented for each lipid. Only phospholipids with acyl chains above 32 carbons and below 41 carbons are represented. Mean and SEM error bars. Two-way ANOVA. A **** p=0.00001, ** p=0.001, **** p=0.000001. B ** p=0.0013, ** p=0.0019, **** p=0.000000001 C **** p=0.000041, ** p=0.009013, * p=0.029 **** p=0.00001 D ** p=0.0016, * p=0.0293, * p=0.0144 E **** p= 0.0000001 * p=0.014. (F) Addition of choline does not rescue the *ptc-3(RNAi)* phenotype n= 68 (no-choline) and 251 (choline). Mean and SEM error bars. (G) *nhr-8(hd117)* animals were treated with *ptc-3(RNAi)* and survival was compared with nhr-8(hd117) NHR-8::GFP expressing animals Mean and SEM error bars. n= 37 (nhr-8(hd117)) and 24 (*nhr-8(hd117)*; NHR-8::GFP). (H) NHR-8 over-expressing animals were treated with *ptc-3(RNAi)* and survival was scored n= 97 (N2) and 114 (NHR-8 OE). Mean and SEM error bars. (I) SBP-1::GFP localization was determined under different RNAi and cholesterol conditions. n=10 Scale bar 5 µm. Statistical source data are available as source data.

Figure S6

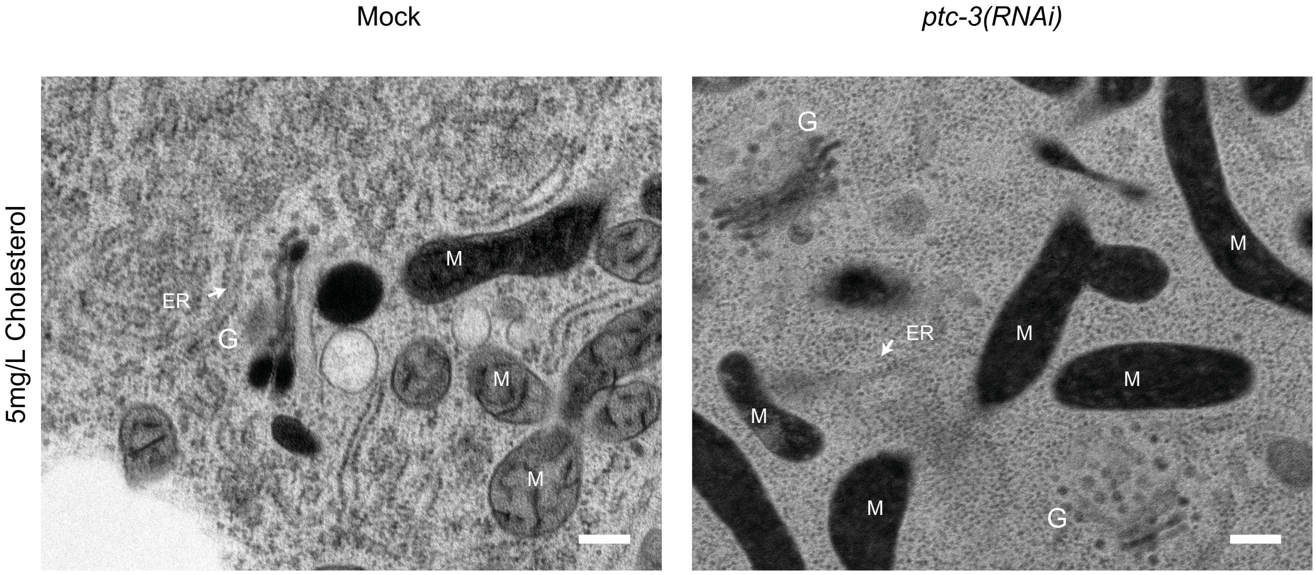

Figure S6. Golgi structure is not dramatically affected in *ptc-3(RNAi)* animals. TEM images showing Golgi structure in Mock and ptc-3(RNAi) treated animals. No major disruption of the Golgi apparatus was observed. ER: Endoplasmic reticulum, M: mitochondria, G: Golgi. Scale bars 200 nm. n= 4 (mock) and 6 *(ptc-3(RNAi))*.

Table S1

Primer name	Sequence
pvha6_fwd	5’-gacggtatcgataagcttgatatcggtatactatttattactcgatacttttg-3’
pvha6_rev	5’-cacgcttgccatttttttatgggttttggtagg-3’
D4H_fwd	5’-aaacccataaaaaatggcaagcgtgagcaag-3’
D4H_rev	5’-ttttgcatttatcttaattgtaagtaatactagatccagggtataaag-3’
tubter_fwd	5’-acttacaattaagataaatgcaaaatcctttcaag-3’
tubter_rev	5’-actagtggatcccccgggctgcaggtgagactttttttcttggc-3’
CH428	5’-gaaaaagagatttggcctactgcagtcgaggaaacccacaaatggcgactatgagtaaaggagaagaacttttca-3’
CH429	5’-gccgaaaaactcgaacttacatttgaaacattgctcggcacactttgacttttgtatagttcatccatgcca-3’
VectorFL_fwd	5’-gctagagctcgctgatcag-3’
VectorFL_rev	5’-ggtggtaccaagcttggg-3’
PTC-3_fwd	5’-gacccaagcttggtaccaccatgaaggtgcattcggaacaac-3’
PTC-3_rev	5’-gctgatcagcgagctctagcttacttgtgcgctggcgatg-3’
nhr41_F	5’-ACGTCGAGTCGTCCACATTT-3’
nhr41_R	5’-TCAGATCTCCCGAGCTCAAT-3’
nhr181_F	5’-TGCGGAACAAAAAGCAGAGC-3’
nhr181_R	5’-ATCTTTGTAGGTTACGTGACCC-3’
cdc42_F	5’-CCTCTATCGTATCCACAG-3’
cdc42_R	5’-GGTCTTTGAGCAATGATG-3’
nhr168_F	5’-GGGAAACTGGCACCAATGAAG-3’
nhr168_R	5’-GTTGCGAGAGGTCAGGCACCG-3’
