# Supplementary material for: Patched regulates lipid homeostasis by controlling cellular cholesterol levels
Source: Nat Commun. 2021 Aug 12;12:4898. doi: 10.1038/s41467-021-24995-9 (PMC8361143; doi:10.1038/s41467-021-24995-9)
Supplement: Supplementary file 3 — Description of Additional Supplementary Files [file 41467_2021_24995_MOESM3_ESM.pdf]

Description of Additional Supplementary Files

File Name: Supplementary Movie 1
Description: Mock ER-3D reconstruction. In a FIB-SEM Z-stack ER was identified by iLastik training. ER structures showed a reticulated morphology. Scale bar 0.4 µm.

File Name: Supplementary Movie 2
Description: ptc-3(RNAi) ER-3D reconstruction. In a FIB-SEM Z-stack ER was identified by iLastik training. Upon ptc-3(RNAi) ER-sheets were identified. Scale bar 0.4 µm.
