# Supplementary material for: Patched regulates lipid homeostasis by controlling cellular cholesterol levels
Source: Nat Commun. 2021 Aug 12;12:4898. doi: 10.1038/s41467-021-24995-9 (PMC8361143; doi:10.1038/s41467-021-24995-9)
Supplement: Supplementary file 6 — Reporting Summary [file 41467_2021_24995_MOESM6_ESM.pdf]

nature research

Corresponding author(s): Anne Spang

Last updated by author(s): Jun 28, 2021

Reporting Summary

Nature Research wishes to improve the reproducibility of the work that we publish. This form provides structure for consistency and transparency in reporting. For further information on Nature Research policies, see Authors & Referees and the Editorial Policy Checklist .

Statistics

For all statistical analyses, confirm that the following items are present in the figure legend, table legend, main text, or Methods section.

n/a	Confirmed	
☐	☒	The exact sample size (*n*) for each experimental group/condition, given as a discrete number and unit of measurement
☐	☒	A statement on whether measurements were taken from distinct samples or whether the same sample was measured repeatedly
☐	☒	The statistical test(s) used AND whether they are one- or two-sided<br>*Only common tests should be described solely by name; describe more complex techniques in the Methods section.*
☒	☐	A description of all covariates tested
☒	☐	A description of any assumptions or corrections, such as tests of normality and adjustment for multiple comparisons
☐	☒	A full description of the statistical parameters including central tendency (e.g. means) or other basic estimates (e.g. regression coefficient) AND variation (e.g. standard deviation) or associated estimates of uncertainty (e.g. confidence intervals)
☐	☒	For null hypothesis testing, the test statistic (e.g. *F*, *t*, *r*) with confidence intervals, effect sizes, degrees of freedom and *P* value noted<br>*Give P values as exact values whenever suitable.*
☒	☐	For Bayesian analysis, information on the choice of priors and Markov chain Monte Carlo settings
☒	☐	For hierarchical and complex designs, identification of the appropriate level for tests and full reporting of outcomes
☒	☐	Estimates of effect sizes (e.g. Cohen's *d*, Pearson's *r*), indicating how they were calculated

Our web collection on statistics for biologists contains articles on many of the points above.

Software and code

Policy information about availability of computer code

Data collection	Software microscopy - ZEN Black 2.1 (LSM 880 Airyscan), ZEN 2.6 (Blue edition; widefield microscope), Western Blots: captured on a FUSION FX Vilber Lourmat using FusionCapt Advance StepOne V2.2.3 Software (Applied Biotechnologies)
Data analysis	Fiji 1. ImageJ version 2.1.0/1.53c and plugins therein (plug-in Lipid Droplet Counter, [https://imagej.net/TrakEM2],<br>Prism 7 and 9, Maps software V3 (Thermo Fisher Scientific, Eindhoven, The Netherlands), iLastik V1.3.0, IMARIS 9.2, StepOne V2.2.3 Software (Applied Biotechnologies). OMERO.web 5.3.4-ice36-b69, Python v3.8.5

For manuscripts utilizing custom algorithms or software that are central to the research but not yet described in published literature, software must be made available to editors/reviewers. We strongly encourage code deposition in a community repository (e.g. GitHub). See the Nature Research guidelines for submitting code & software for further information.

Data

Policy information about availability of data

All manuscripts must include a data availability statement. This statement should provide the following information, where applicable:
- Accession codes, unique identifiers, or web links for publicly available datasets
- A list of figures that have associated raw data
- A description of any restrictions on data availability

The authors declare that the data supporting the findings of this study are available within the article and source data files. Lipidomics data are available at https://lipidomes.epfl.ch/exps/2243 , https://lipidomes.epfl.ch/exps/2244 and https://lipidomes.epfl.ch/exps/1709 . Lipidomics de-isotoping data code is available from DOI:10.5281/zenodo.5032252 All other data supporting the findings of this study are available from corresponding author upon request.

Field-specific reporting

Please select the one below that is the best fit for your research. If you are not sure, read the appropriate sections before making your selection.

☒ Life sciences ☐ Behavioural & social sciences ☐ Ecological, evolutionary & environmental sciences

For a reference copy of the document with all sections, see nature.com/documents/nr-reporting-summary-flat.pdf

Life sciences study design

All studies must disclose on these points even when the disclosure is negative.

Sample size	No sample size was determined. Sample size was chosen in each case according to experimental design. Cell biology experiments were performed at least in 3 independent biological repeats. For C. elegans experiments, worms analyzed were from at least 3 independent replicates. Exact numbers are given in the figure legends.
Data exclusions	Animals that move during live imaging acquisition were excluded from the analysis.
Replication	At least 3 independent experiments were performed per condition with exception of FIB-SEM analysis, the experiment was a validation of the TEM analysis therefore only 1 animal per condition was analyzed which validated the observations from the 2D images. All experiments were shown to be reproducible.
Randomization	All animals were pulled together and randomly split into experimental groups.
Blinding	No blinding was required since animals characteristics were quantitatively evaluated. Furthermore, animals with strong phenotypes as lethality are easily spotted during data acquisition making blinding impossible

Reporting for specific materials, systems and methods

We require information from authors about some types of materials, experimental systems and methods used in many studies. Here, indicate whether each material, system or method listed is relevant to your study. If you are not sure if a list item applies to your research, read the appropriate section before selecting a response.

Materials & experimental systems

n/a	Involved in the study	
☐	☒	Antibodies
☐	☒	Eukaryotic cell lines
☒	☐	Palaeontology
☐	☒	Animals and other organisms
☒	☐	Human research participants
☒	☐	Clinical data

Methods

n/a	Involved in the study	
☒	☐	ChIP-seq
☒	☐	Flow cytometry
☒	☐	MRI-based neuroimaging

Antibodies

Antibodies used	Peptide rabbit antibodies against C. elegans PTC-3 were generated for this study by Eurogentec with peptides SASHSSDDESSPAHK and EVRRGPELPKENGLG. Alexa Fluor 488-goat anti-rabbit IgG (H+L) (Invitrogen; A-11034), Monoclonal Anti-α-Tubulin clone B-5-1-2, T5168 Sigma-Aldrich, HRP-coupled antibodies goat anti-Mouse IgG (H+L) (ThermoFisher scientific; 31430) or polyclonal HRP-conjugated goat-antirabbit IgG (ThermoFisher scientific; 31460)
Validation	Most antibodies are commercially available and validated by the manufacturer. In addition, most of the antibodies have been used by other research groups previously as referenced in the associated manuscript. Antibodies against PTC-3 were validated by Immunoblot of mock and ptc-3 (RNAi) treated worms.

Eukaryotic cell lines

Policy information about cell lines

Cell line source(s)	HEK 293 from ATCC
Authentication	standard cell line, authenticated by ATCC.
Mycoplasma contamination	We routinely test our cell lines for mycoplasma contamination. All cell lines were negative

Commonly misidentified lines (See ICLAC register)	No commonly misidentified cell lines were used

Animals and other organisms

Policy information about studies involving animals; ARRIVE guidelines recommended for reporting animal research

Laboratory animals	C. elegans, hermaphrodite animals were used for all the analysis. All animals were analyzed after 3 days of culture (adults) unless specified different. The following strains were used: N2, nhr-49(nr2041) [ges1p::3xHA::nhr-49(cDNA)::unc-54 3'UTR + myo-3p::mCherry::unc-54 3'UTR], kbIs7 [nhx-2p::rde-1 + rol-6(su1006)], sbp-1(ep79) [sbp-1::GFP::SBP-1; rol-6(su1006)], and nhr-8(hd117). Strains [vha-6p::YDA::mCherry::tub 3'UTR + sur-5p::GFP], [vha-6p::YQDA::mCherry::tub 3'UTR+ sur-5p::GFP], and ptc-3::gfp [WRM064cC06::GFP+ sur-5p::GFP]+pMF435:Ppgp-1::mCherry::unc-54 3'UTR were generated for this study.
Wild animals	no wild animals were used in the study.
Field-collected samples	no field collected samples were used in the study
Ethics oversight	Non-vertebrate model system. No ethical oversight required.

Note that full information on the approval of the study protocol must also be provided in the manuscript.
